# Application-Aware SDN-Based Iterative Reconfigurable Routing Protocol for Internet of Things (IoT)

**DOI:** 10.3390/s20123521

**Published:** 2020-06-22

**Authors:** Ayesha Shafique, Guo Cao, Muhammad Aslam, Muhammad Asad, Dengpan Ye

**Affiliations:** 1 School of Computer Science and Technology, Nanjing University of Science and Technology, Nanjing 210094, China; ayeshashafique@njust.edu.cn; 2 School of Cyber Science and Engineering, Wuhan University, Wuhan 430070, China; aslamhayat@whu.edu.cn (M.A.); yedp@whu.edu.cn (D.Y.); 3 Department of Computer Science, Nagoya Institute of Technology, Nagoya 466-8555, Aichi, Japan; m.asad@itolab.nitech.ac.jp

**Keywords:** software-defined networking, Internet of Things, network reconfiguration, application-aware, heterogeneity-aware

## Abstract

The central intelligence offered by Software Defined Networking (SDN) promise the smart and reliable reconfiguration which enables the scalability of dynamic enterprise networks. The decoupled forwarding plane and control plane of SDN infrastructure is a key feature that supports the SDN controller to extract the physical network topology information at runtime to formulate network reconfigurations. This SDN-based network reconfiguration enables application-aware routing capability for Internet of Thing (IoT). However, these IoT enabled SDN-based routing protocols face some performance limitations in iterative reconfiguration process due to complete centralized path selection mechanism To this end, in this paper, we propose SDN-Based Application-aware Distributed adaptive Flow Iterative Reconfiguring (SADFIR) routing protocol. The proposed routing protocol enables the distributed SDN iterative solver controller to maintain the load-balancing between flow reconfiguration and flow allocation cost. In particular, the proposed routing protocol of SADFIR implements multiple SDN controllers that collaborate with network devices at forwarding plane to develop appropriate clustering strategy for routing the sensed information. This distributed SDN controllers are assisted to clustering topology that successfully map the residual network resources and also enable unique multi-hop application-aware data transmission. In addition, the proposed SADFIR monitor the iterative reconfiguration settings according to the network traffic of heterogeneity-aware network devices. The simulation experiments are conducted in comparison with the state-of-the-art routing protocols which demonstrates that SADFIR is heterogeneity-aware which is able to adopt the different scales of network with maximum network lifetime.

## 1. Introduction

**Figure 1:**
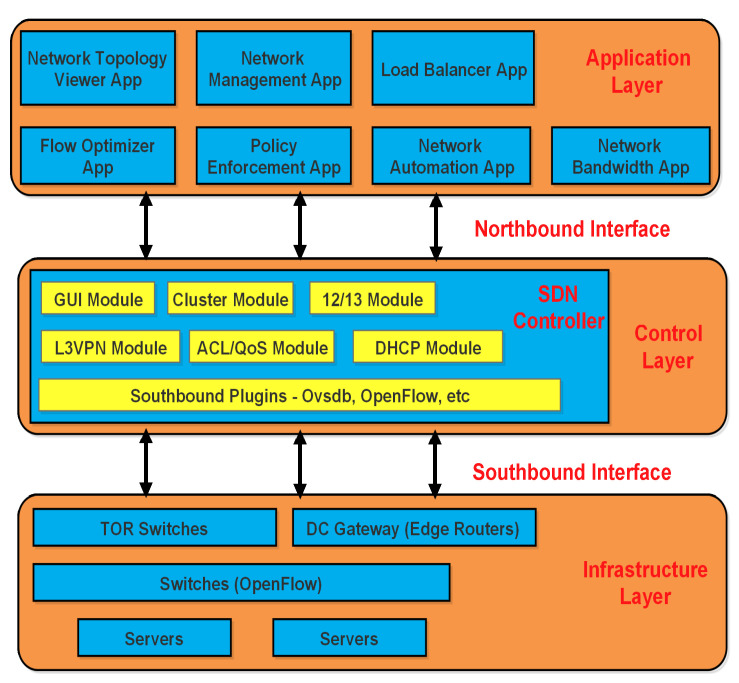
The General Architecture of Software Defined Network (SDN) [9].

The main technical advancements offered by Software Defined Networking (SDN) provides the unique separation of the control plane from forwarding plane in the hybrid SDN-based WSNs oriented Internet of Things (IoT) architectures [1]. IoT is advanced and heavily deployed Information and Communication Technology (ICT) application for modern-day communication systems. Some applications of IoT utilize the thousands of sensor nodes, that are organized by complicated wired and wireless environments to develop the information collection networks [2,3]. Meanwhile, the advanced networking settings of SDN allow central control over deployed IoT sensor nodes by the support of the SDN central controller. This central control, enable the SDN controller to act as a Network Operating System (NOS), which maintains a range of exceptional central control functions that include network topology to upper layer behaviour of the network. Network topology presents the nodes physical localization to wireless connectivity settings for deployed sensor nodes and their respective report transmission tasks within the data plane arrangements [4,5]. Whereas these data plane tasks are centrally monitored and controlled by the central SDN controller at the control plane, this new primary controller interventions perform network management, which allows central network reconfigurations by global systems settings at SDN controller [6]. The SDN-based WSNs oriented IoT architecture basically introduce a new dimension of application-sensitive and heterogeneity-aware WSNs. These settings bring stability and offer alternative solutions for existing distributed WSNs network configurations [7]. These SDN-enabled IoT integration solutions alter the management systems of WSNs oriented IoT and offer practicality at the commercial level. In the general architecture of SDN-based WSN as shown in Figure 1, the very first requirement is to have the support of SDN in the WSN architecture [8].

## 2. Characteristics of SDN Architectures:

The exiting SDN-Based WSNs architectures are divided into the following categories:

- Distributed SDN-based WSNs Architecture: Although SDN-based WSNs are developed to achieve the centralized control but here, the distributed SDN-based WSNs architectures means the network architectures that contain a single controller and complete absence of distributed controllers. The distributed SDN-based WSNs architectures provide multiple SDN controllers to be deployed within WSNs and perform over central networking behaviour by shifting the control plane functionality to SDN controllers. These multiple controllers react as clusters leading nodes and take responsibilities of SDN-based CHs. These SDN controllers are rich in computational and energy resources and offer relief to normal sensor nodes by skipping complex computations [10,11]. Overall combinations of these multiple SDN-controllers maintains a logical integrated network global view and implement the network reconfigurations to execute the sophisticated traffic engineering for WSNs. At the earlier stages of research on SDN-based WSNs architectures, only a few solutions were available, which allows the distributed deployment of multiple SDN controllers otherwise, the majority of existing solutions offers only a single centralized SDN controller to maintain the central control. The design and implementation of communications protocols for complex distributed clustering SDN-based WSNs model is still an open challenge. Some of the state-of-the-art distributed SDN-based WSNs architectures are SDWN, Sensor OpenFlow and Mininet-WiFi [12,13,14].
- Centralized SDN-based WSNs Architecture: This type of network model depicts an SDN-based WSN architecture enabling central network configurations with the help of the SDN controller, which maintains the overall record of the whole network that results in the efficiency of network management. These centralized SDN-based solutions manage dictations over sensor nodes with multiple sensing units to prioritized the application-sensing and active modes’ participation according to their residual energy resources. QoS and security enhancement are other critical features for SDN-based WSNs architectures achieved at the control plane while enabling SDN integration at all levels of the design. Smart and CRLB are examples of centralized SDN-based WSNs Architecture [15].
- Hybrid SDN-based WSNs Architecture: The hybrid SDN-based WSNs Architecture is designed to achieve flexibility and cost-effectiveness at the same time. These architectures consist of few controllers deployed within the network for long distant nodes while these networks can manage networking responsibilities, even the failure of distributed controllers. This architecture can behave fully centralized at the time to carry on SDN-based WSNs networking architecture. Some of the hybrid management systems include Sensor Network Management System (SNMS) and Wireless Sensor Network Management System (WSNMS) [16].
- Mobility Management SDN-based WSNs Architecture: The new application spectrum of conventional WSNs contains many networking solutions for mobility-based networks. These networks are facing more connectivity and central management control as compared to static WSNs. In the management of sensor nodes mobility, while maintaining a global view of reasonable network topology, the SDN-controller needs to support node mobility, which consists of unreliable wireless links and continuous reconfigurations of routing traffic engineering [17]. The sensor nodes in the SDN-based WSNs are capable of mobility that helps in packets transmission and task execution, which makes it obligatory to monitor and manage the particular movement of sensor nodes in the entire network. Mobility management of SDN-based WSNs architectures also needs to deal with high mobility nodes entering and leaving the networks. To this end, many research contribution has conducted on mobility management of SDN controller based Wireless Cellular Network [18].

The exiting distributed SDN-based WSNs architectures perform better than complete centralized SDN-based architectures. However, these distributed architectures have limitations as clustering decisions still solely reside at deployed multiple SDN controllers. In this paper, we proposed the SDN-Based Application-aware Distributed adaptive Flow Iterative Reconfiguring (SADFIR) routing protocol to extend the distributed property of clustering formulation by engaging the network nodes of Internet of Things(IoT). The major contributions of this paper are the following:

- The proposed SADFIR routing protocol computes the network topology with multiple SDN-controllers at control layer and computes the residual resources to save the computational cost of IoT devices.
- At the initial stage of proposed SADFIR, the SDN controllers select the set of most eligible IoT nodes to be selected as Cluster-Heads (CHs) and then multicast the notification to eligible nodes to further proceed the distributed cluster formulation.
- At the final stage of cluster formulation, the proposed SADFIR performs distributed selection of final CHs at infrastructure layer of deployed IoT nodes. This extendedly distributed property of SADFIR routing protocol provides the novelty to our proposed routing protocol.
- The proposed SADFIR routing protocol manages the heterogeneity-aware distributed cluster formation of heterogeneous network. Furthermore, the application-awareness property of the proposed model enables the IoT nodes to transmit the sensed information of multiple applications.

The rest of the paper is organized as follows: Section 2 explains the literature review and provide a comparative view of the state-of-the-art approaches. In Section 3, we precisely explain the heterogeneous network model of the proposed SADFIR. In Section 4, we propose the SADFIR routing protocol and explains all the phases in detail. We define the simulation parameters and evaluate the proposed SADFIR in Section 5. We draw the conclusion of this paper in Section 6.

## 3. Related Work

In existing parallel literature of WSNs, the design of internet of things (IoT) is constructed in which hundreds and thousands of sensor nodes perform suitable interconnection to proceed the sensing and monitoring processes. As, existing conventional architectures of WSNs inherent many challenges due to the fragile distributed management system, similarly IoT face identical issues of nodes resource constraints of heterogeneity-limitations and application-awareness [19]. These issues are highlighted by the widening of the recent market for sophisticated applications at a larger scale networking range of density. Conventional WSNs struggles as deployed nodes are not reprogrammable, which makes nodes responsible for the basic operations such as data forwarding and network control, and this makes the node behave ultimately like an autonomous system [20]. These technical limitations result in a lack of cognitive flexibility to manage advanced-level quality application-awareness. With the development of effective WSNs networking new mechanisms, it is essential to promote the central management systems for maintenance and resource-effective connectivity. The system needs to be continuous variation in reconfigurations of nodes with a limited lifetime. New configuration settings of WSNs faces this challenge where the data packets and the control packets are transmitted through the existing network bandwidth [21,22].

Industrial demands for a better success rate of IoT insists the researchers to investigate the opportunities promised by SDN proposed solutions to develop flexible management of IoT that separate the control logic from the network nodes or actuators. These advanced features of SDN-based central management of WSNs architectures are attractive enough to gain researchers’ attention as SDN-based management systems are being developed for every type of mainframe networks [23]. SDN-based central management systems provide immense flexibilities to develop and exercise the overall network management protocols and their applications. Consequently, the IoT nodes are capable of reprogrammable in case of priority operations [24]. To this end, the various researchers investigate and proposed solutions to manage the IoT networks parallel to WSNs, and some of these solutions are designed through the (SDN).

In contrast with conventional WSNs management systems, SDN-based WSNs offer real-time reconfigurations to achieve network configuration optimization and application-sensitive environmental sensing. Consequently, new traffic engineering protocols need to be developed to support real-time reconfigurations to utilize application-specific platform-independent central control with the help of SDN central controller. The evolution of SDN-based WSNs architecture is a recent development, and advanced protocols are under construction [25]. In light of resource constraints of WSNs, routing protocols are being proposed to offer SDN-based WSNs inter-networking. The available routing protocols which are introduced in the past few years are discussed below:

- Situation-Aware Routing Protocol Switching for SDN-based WSNs: In [26], Situation-aware routing protocol switching scheme has been introduced, which design a platform-independent architecture to implement multiple routing protocols for SDN-based WSNs. This situation-aware switching mechanism allows the network operators to not only reconfiguration of application-specific sensing attributes but also provide flexibility to change routing protocol in real-time. This SDN-based WSNs routing modeling solution divides its working into phases; in the initial stage, central control compute network resources according to current network topology and decide for specific routing protocol should be implemented. Whereas, in the second phase, the central controller performs reconfiguration of sensor nodes according to selected routing protocol and dictates nodes to report according to the current network settings. Although this routing switching environment is a valuable contribution for SDN-based WSNs solutions, this scheme just considering the existing state-of-the-art routing protocols such as AODV, DSDV, LSR, and DLSR, this solution lacks the proposal of novelty routing protocol; meanwhile, AODV, DSDV, LSR, and DLSR routing protocols are not designed for SDN-based WSNs, so the required performance is still a challenge for this solution.
- Non-linear Weight Particle Swarm Optimization (NWPSO) algorithm-based routing protocol: In [27], the authors proposed a routing protocol based upon Non-linear Weight Particle Swarm Optimization (NWPSO) algorithm to implement centralized multi-tasking for SDN-based WSNs (SDWSNs). This protocol develops the clustering mechanism of selecting suitable controllers from the sensor nodes with maximum residual energy, and these controller nodes are responsible for collecting reports at the intra-cluster communication level. NWSPO algorithm functionality is utilized to select the most suitable cluster’s controller to assign multi-tasking and inter-cluster communication. The association phase of this routing protocol is similar to conventional routing protocols of WSNs. This routing protocol shows performance betterment than LEACH routing protocol, but it lacks to propose classical SDN penetration in WSNs.
- QoS-aware routing mechanism for OpenFlow-enabled WSNs: In [28], a unique QoS-aware routing mechanism for OpenFlow-enabled WSNs routing protocol is proposed. Similar to NWPSO, this proposed model also selects some OpenFlow to enable nodes as intermediate nodes to transmits network reports to BS. This routing protocol enhances QoS by choosing the most strong links called a feasible path and then also define the best effort paths. In most of the cases, it is supposed that possible ways will carry transmissions. In case of any unexpected failures of feasible paths, the best effort paths will carry on the network operation. If no path satisfies the required QoS, the road will be decided by the proposed algorithms depending on flow types: delay-sensitive, bandwidth-sensitive, and best-effort traffic. This proposal also only provide an abstract footprint for SDN-based WSNs and also highlight future restrictions.
- SDN Enabled SPIN Routing Protocol: In [29], the SDN-enabled SPIN routing protocol is proposed for SDN-based WSNs. This SDN-enabled SPIN routing protocol introduces SDN-enabled sensor nodes and divides into normal and controller nodes. The SDN-enabled reconfigurations work behind the scene of the SPIN routing protocol. This proposed model claims to achieve better energy efficiency and also enhance network security by strengthening the central control over WSNs. This protocol also lacks the envision of the central controller at BS as individual nodes contain limited networking resources.

In above mentioned routing protocols, two of the major drawbacks are lack of application-awareness and distributed nature cluster-formulation. In order to achieve these two distinct features, we propose SDN-based Application-aware Distributed adaptive Flow Iterative Reconfiguring (SADFIR) routing protocol for IoT. In particular, the SADFIR improves the existing features and characteristics of SDN-based WSNs, which is summarized as a comparative view in Table 1.

## 4. Heterogeneous Network Model of Proposed Model

In this section, we enhance the SACFIR network architecture of SDN-enabled Iteratively Reconfigurable WSNs (SDN-IRWSNs) [9] to Distributed SDN-enabled Iteratively Reconfigurable Internet of Things (DSDN-IRIoT). This network design of DSDN-IRIoT extends the suitability of the proposed SADFIR routing protocol for the Internet of Things.

### 4.1. Distributed SDN-Enabled Iteratively Reconfigurable Internet of Things (DSDN-IRIoT)

**Figure 2:**
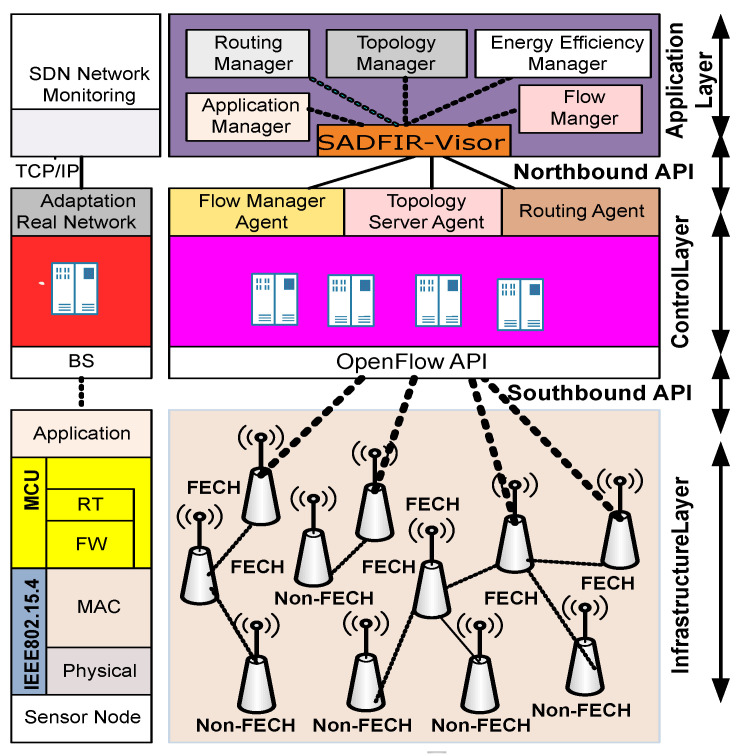
Hybrid architecture of heterogeneous SDN-IRWSNs.

The proposed network model of DSDN-IRIoT is equipped with a distributed layer of multiple SDN controllers at the control layers which actively collect network key information from OpenFlow enabled IoT devices at the forwarding layer. The multiple SDN controllers at the control layer provide distributed clustering coordination, and network scalability is available due to additional network resources of the control layer as control layer is coupled within Base Station (BS), so it drastically reduces the computational and energy resources bourdon of forwarding layer devices. The network scalability offers additional network applications for dense network resources. The abstract of the network architecture of DSDN-IRIoT is shown in Figure 2.

Mainly, the SDN enabled network architecture consists of the following layers; infrastructure layer, control layer, and application layer. Meanwhile, every unique layers set of communication protocols and collectively formulate the effective open standard protocol stack. Precisely, the application layer consists of SADFIR-Visor that supervises the information collected from the control and infrastructure layer and coordinates with different application layer APIs. The programmability of application layers APIs generates the expected network response from northbound layers, and SADFIR-Visor handovers the response to designated network controllers. Similarly, at the protocol stack level of the application layer, Inter-Networking Processing (INP) protocol utilizes a path selection algorithm that instructs the controller to dictate specific routing schemes at the infrastructure layer. In this paper, we propose a SADFIR routing algorithm to establish a path selection mechanism. The SADFIR formulates the periodic route reconfiguration mechanisms on behalf of the periodically retrieved information from the control and infrastructure layer. Link Layer Discovery Protocol (LLDP) is a crucial protocol to collect network topology and network heterogeneity. At the control layer, multiple controller agents like; flow manager agent, topology server agent and routing agent actively communicate with northbound and southbound APIs. Similarly, the infrastructure layer consists of smart IoT devices. These intelligent IoT devices are equipped according to the application of the network, but some of the components are essential for all the accessories such as; Sensing Unit (SU) and Power Unit (PU) which accumulatively makes a Micro Controller Unit (MCU) that maintains the basic operations of IoT device with limited computational capacity. As SDN-enabled IoT devices are equipped with OpenFlow, so these devices are designed to have insufficient memory to maintain the forwarding tables generated by SDN controllers.

### 4.2. DSDN-IRIoT Infrastructure Layer Network Modeling

The initial settings of the infrastructure layer of the DSDN-IRIoT network model defines the pattern of periodic reconfigurations requirements throughout network operation. In the initial configuration, BS is located at the network outer boundary. Whereas, the DSDNs controllers resides at the BS with the assumption of extensive heterogeneity and processing capacity. Meanwhile, IoT devices are scattered with different heterogeneity levels and categorized into normal nodes, advanced nodes, and super nodes with ascending energy order.

## 5. Proposal of SADFIR

In this section, we propose the SDN-Based Application-aware Distributed adaptive Iterative Flow Reconfiguring (SADFIR) routing protocol to configure the DSDN-IRIoT network model periodically. The principal purpose of this periodic routing reconfiguration of the proposed SADFIR protocol is to manage heterogeneity-awareness and application-sensitivity of sensed information. Consistency of periodic reconfigurations sometime results in more computational overhead and reduces the duration of actual data transmissions. In order to avoid the computational cost, the proposed SADFIR model optimizes the reconfigurations by delaying the periodic execution up to 10th round, if the heterogeneity index of IoT devices remains stable. Distributed SDN controllers follow the unique computational threshold to enable reconfiguration methodology for the current period;

(1) $$\begin{array}{c} RM_{q}=\left\{\begin{array}{cc} \frac{D_{p}}{\left(1-D_{p}\times (qmod\frac{1}{D_{p}}\right)}, & \mathrm{if}rE>0\\ 0, & \mathrm{otherwise} \end{array}\right. \end{array}$$

where, $RM_{q}$ is threshold settings for reconfiguration methodology for current round *q*, while $D_{p}$ is desired percentage, $D_{p}=0.1$ represents the initial default settings of desired percentage, rE>0 indicates that computational criteria is implementable on alive nodes. This threshold methodology provides additional heterogeneity-level network optimization to establish a smart reconfiguration strategy.

**Figure 3:**
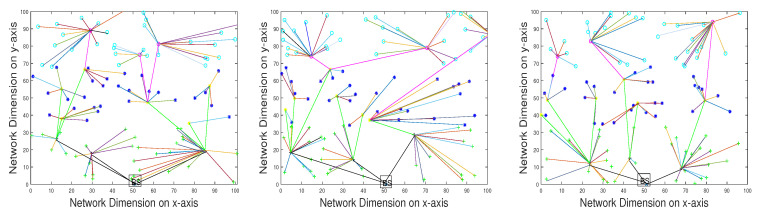
Multiple iterations of clustering topology in SADFIR.

**Figure 4:**
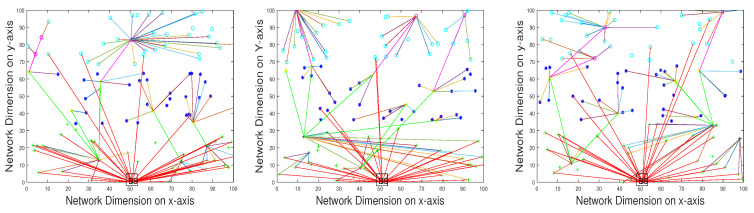
SADFIR routing mechanism and protocol.

**Figure 5:**
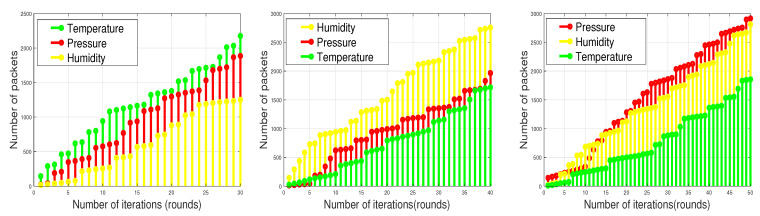
Application-aware and environment-sensing of SADFIR routing protocol.

**Figure 6:**
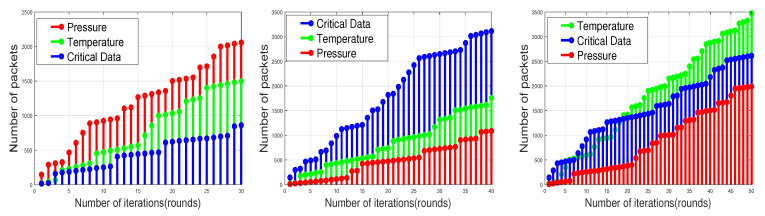
Direct transmission of critical data in SADFIR routing protocol.

The execution of SADFIR routing protocol generates the clustering formulation, which is shown in Figure 3 and Figure 4. In particular, the Figure 3 and Figure 4 provide slightly different data transmission as SADFIR is flexible to report critical information directly. The above described DSDN-IRIoT network model consists of IoT enabled sensors that provide sensing ability of multiple applications such as; temperature, humidity, and pressure. Figure 5 shows the communication of different sensors transmitting data of the various applications. Similarly, Figure 6 indicates the SADFIR ability to communicate the critical data directly once appeared in the sensing of IoT network environment.

The complete implementation of the proposed SADFIR is divided into three phases of interrelating processes called; Network Topology Management Phase (NTMP), Network Settling Phase (NSP), and Network Forwarding Phase (NFP). The technical details of these phases are given in the next three subsections.

### 5.1. SADFIR Network Topology Management Phase

The proposed SADFIR routing algorithm formulates unique coordination between infrastructure layer network topology and application layer path selection re-computation to develop an industrial level solution. The re-computation to establish reconfiguration of network topology demands periodic updates through the topology discovery mechanism. More comprehensive network updates assist the SADFIR routing algorithm in generating precisely accurate policy responses from the upper layer and distributed SDN controllers play a vital role in managing these computational tasks. The distributed SDN controllers follow network topology discovery through the state-of-the-art SDN controllers like POX and NOX SDN controllers [36,37]. The OpenFlow Discovery Protocol (OFDP) is implemented with a unique design extension in IoT based networks. These OFDP coordinate with the existing Link Layer Discovery Protocol (LLDP) [38] to manage these tasks at both southbound and northbound. The coordination of OFDP and LLDP is generally referred to as Topology Discovery (TD).

The proposed SADFIR utilizes these TD hello messages to extract particular network information at infrastructure layer such as; nodes residual energy, distance to BS, a mutual distance of nodes from previous Cluster-Head (CH), epoch information, RSSI level among neighbors and ID of serving SDN controller. This critical information is responded by IoT devices to TD iterative broadcasting by distributed SDN controller at BS. IoT network devices have heterogeneous residual energy resources; that is why distributed SDN controllers need to collect the information from all alive nodes and share to understand the status of the nodes. If a consolidated database of all distributed SDN controllers misses any entry of previous rounds entry table, then it is considered as a dead node. Conventional static network environments utilize the TD messages mentioned above, otherwise in mobility-based scenarios that demand more frequent localization information. The frequency of TD exchange rate in a static environment depends upon the duration of one period. The SADFIR-Visor holds the authority to alter the TD interval rate, and SADFIR-Visor manages this iteration according to the scalability of the network.

### 5.2. Network Settling Phase (NSP) of SADFIR

The network settling phase primarily computes clustering reconfiguration of IoT devices given network topology and coordination of SDN controllers with SADFIR-Visor. The selected clustering arrangement delivers routing responsibility of sensed information. The heterogeneity-awareness is a significant consideration of the proposed SADFIR routing algorithm to utilize residual network resources efficiently. Application-awareness is the second most critical challenge for the proposed model to deliver maximum actual sensing signals instead of engaging network resources in redundant computational tasks. To achieve higher throughput of application-aware data, the proposed model deliberately delays the reconfiguration process up to 10th rounds.

Precisely, distributed SDN controllers collect the network heterogeneity and application-awareness details from network topology during the NTMP phase. The Network Settling Phase (NSP) utilizes the network topology view to compute the average energy of residual network resources of IoT devices by the following equation:

(2) $$\bar{RE_{i}\left(s\right)}=\frac{1}{N}\sum_{i=1}^{N}RE_{i}\left(s\right)$$

where *N* represents the IoT devices and *s* represents the network resources, which accumulates $\bar{RE_{i}\left(s\right)}$ and represents average energy of residual network resources, whereas individual IoT device contains $RE_{i}\left(s\right)$ as residual energy.

Different distributed SDN controllers correspond to different IoT devices for all the alive nodes of (1≤i≥N), that is why all these SDN controllers share the database at BS after computation of ability to lead the potential cluster. Initially, SDN controllers set the basic criteria of a leading role for Cluster-Head (CH) to contain residual energy higher than the average energy of the network region, which is denoted as $\left(E_{i}\geq \bar{RE_{i}\left(s\right)}\right)$. Those IoT devices which meets the criteria of $\left(E_{i}\geq \bar{RE_{i}\left(s\right)}\right)$ are selected as Expected Forwarding Device CHs (EFDCHs). Furthermore, distributed SDN controllers extend the investigation to select better CH by calculating the mutual distance factor of EFDCHs (1≤j≥EFDCHs) with neighbor nodes and BS. The following equation is utilized to calculate the minimum distance with BS:

(3) $$Min-dist_{toBS}{=}^{\sqrt{{\left(X-xj\right)}^{2}+{\left(Y-yj\right)}^{2}}}$$

where $Min-dist_{toBS}$ represents the minimum distance between BS and jth EFDCHs, while xj and yj are location coordinates of jth EFDCHs and *X* and *Y* are the location coordinates of BS. SDN controllers utilize the graph theory [39] to maximize the centrality of EFDCHs in potential cluster formulation. The centrality computation defines the parameters of nodes value and centrality degree and optimizes the process by selecting the highest degree centrality. The nodes from EFDCHs develop the indexing rate of Clustering Centrality (CC) to achieve suitable positioned EFDCHs. Afterwards, CC is computed as an average ratio by which a source S neighboring non-FFDCHs needs to pass through a specific node EFDCH to reach the destination (D) which can be calculated as:

(4) $$CC_{EFECH}=\sum_{S\neq EFECH\neq D}\frac{{}_{SD}\left(EFECH\right)}{{}_{SD}}$$

where ${}_{SD}$ represents the possible number shortest path between two communicated IoT devices, meanwhile ${}_{SD}\left(EFECH\right)$ represents the shortest path which cross-sectioning the calculated EFDCHSs. In this way, distributed SDN controllers derive the suitable Centrally Cluster EFDCHs $CC_{EFDCHs}$, which is a highly influenced IoT device and accommodates all potential cluster members. $CC_{EFDCHs}$ nodes with $\left(E_{j}\geq \bar{RE_{j}\left(s\right)}\right)$ gets the higher ranking indexing at controller layer to be selected as CHs. As our proposed SADFIR has an additional distributed layer at the forwarding infrastructure layer, so distributed SDN controllers broadcast the potential $CC_{EFDCHs}$ nodes to continue the final distributed selection procedure. The potential $CC_{EFDCHs}$ utilize the following formula to select the most suitable CH:

(5) $$\begin{array}{c} SCC_{EFDCHs}=\left\{\begin{array}{cc} \frac{P_{j}}{1-P_{j}*\left(rmod\frac{1}{P_{j}}\right)\times d} & \;\mathrm{if}n\in CC_{EFDCHs}\\ 0 & \;\mathrm{otherwise} \end{array}\right. \end{array}$$

where $SCC_{EFDCHs}$ represents most suitable $CC_{EFDCHs}$, $P_{j}$ is desired percentage of final CHs and the value of $P_{j}=0.3$. While $n\in CC_{EFDCHs}$ represents the group of IoT devices from which $SCC_{EFDCHs}$ are being selected. Finally, $SCC_{EFDCHs}$ advertise the leading status and all $non_{EFDCHs}$ select the best CHs based on the criteria of RSSI and minimum distance.

The SADFIR repeats these reconfigurations formulations throughout the network operations in every round. Distributed SDN controllers optimize the clustering formulation by computing the network analytical cost over 10th consecutive rounds and selects the most economical setting for network reconfiguration. In-network settling phase, one major feature of the proposed method is to evenly distribute the communication load of IoT devices with limited energy and communication resources.

The optimization of communication load of networks *N* clustered IoT devices include two major intra-cluster communications and inter-cluster communications responsibilities. Member devices of cluster initialize *f* report with $f\epsilon \left[1,F\right]$ sensed reports, while the basic assumption defines application type supported by the specific application. Member node *i*lth with *f* type application reports denoted as $\Gamma_{if}$ to selected *j* leading node of $SCC_{EFDCHs}$, while whole collected data from cluster members represented as $\Psi_{jf}$. Meanwhile specific cluster collection is denoted as $\sum_{j=1}^{N}\Psi_{jf}=1$. While intra-cluster communication cost is represented by $Z_{ij}$, and inter-cluster communication is denoted as $Z_{js}$. As distributed SDN controllers successfully fetch the network topology statistics by link layer protocols, so proposed model utilizes the computational ability to calculate analytical consumption of residual energy resources for different possible reconfiguration settings for the consecutive epoch of rounds. Let $\varrho_{f}$ to produce binary variable taking values 0 and 1 to report sensing value generation at the source node. So, the intra-cluster communication cost is calculated by the following equation:

(6) $$Ira-C-Cost=\sum_{f=1}^{F}\sum_{i=1}^{N}\sum_{j=1}^{N}Z_{ij}\Gamma_{if}\Psi_{jf}\varrho_{f}$$

Similarly inter-cluster communication cost of $SCC_{EFDCHs}$ is calculated by:

(7) $$Ier-C-Cost=\sum_{f=1}^{F}\sum_{j=1}^{N}l\varrho_{f}Z_{js}\Psi_{jf}$$

The overall network total communications cost is calculated as:

(8) $$Total-C-Cost=Intra-C_{C}ost+Inter-C_{C}ost$$

(9) $$Total-C-Cost=\sum_{f=1}^{F}\sum_{i=1}^{N}\sum_{j=1}^{N}Z_{ij}\Gamma_{if}\Psi_{jf}\varrho_{f}+\sum_{f=1}^{F}\sum_{j=1}^{N}l\varrho_{f}Z_{js}\Psi_{jf}$$

(10) $$Total-C-Cost=\sum_{f=1}^{F}\sum_{j=1}^{N}\Psi_{jf}\left(l\varrho_{f}Z_{js}+\sum_{j=i}^{N}Z_{ij}\Gamma_{if}\right)$$

where right side of the equation is constant for any node *i* to transmit *f* reports through *j*$SCC_{EFDCHs}$, thus it can be replaced by constant:

(11) $$Total-C-Cost=\sum_{f=1}^{F}\sum_{j=1}^{N}\varsigma_{jf}\Psi_{jf}$$

The distributed SDN controllers coordinates with each other to minimize the cost which can be represented by the following inequalities:

(12) $$\begin{array}{c} Min-Total-C-Cost=\sum_{f=1}^{F}\sum_{j=1}^{N}\varsigma_{jf}\Psi_{jf} \end{array}$$

(13) $$\begin{array}{c} subject-to=\sum_{j=1}^{N}\Psi_{jf}=1,\forall f\epsilon \left[1,0\right] \end{array}$$

(14) $$\begin{array}{c} \Psi_{jf}=0,1\forall j\epsilon \left[1,N\right] \end{array}$$

The above problem description holds the property of binary integer program, and it contains the class NP-Hard complexity in general. For these problems, greedy algorithm executions are needed to experiment with all possible solutions testing every combination. At this particular instance, we focus on processing capabilities by specific quantitative values, which are denoted by Fmax. Now SDN controller’s analytical operation will be more restricted;

(15) $$\begin{array}{c} Min-Total-C-Cost=\sum_{f=1}^{F}\sum_{j=1}^{N}\varsigma_{jf}\Psi_{jf} \end{array}$$

(16) $$\begin{array}{c} subject-to=\sum_{f=1}^{F}\Psi_{jf}\leq Fmax\forall j\epsilon \left[1,N\right] \end{array}$$

(17) $$\begin{array}{c} \sum_{j=1}^{N}\Psi_{jf}=1,\forall f\epsilon \left[1,0\right] \end{array}$$

(18) $$\begin{array}{c} \Psi_{jf}=0,1\forall j\epsilon \left[1,N\right] \end{array}$$

This settling phase of SADFIR routing algorithm achieves effective cost reduction and results in prolong network lifetime by introducing the most economical utilization of residual resources. In the algorithm below, we represent the functionality of the settling phase of proposed SADFIR. In particular, in Algorithm 1, *N* number of sensor nodes are deployed in the network where, SADFIR selects FECHs and non-FECHs. The whole criteria for selecting the FECHs and non-FECHs is divided into 5 conditions proved in the equations above.

### 5.3. Network Forwarding Phase (NFP) of SADFIR

After settling the reconfigurations of forwarding rules for FECHs and non-FECHs in NSP, the Network Forwarding Phase (NFP) initiates the actual communication for traffic transportation in the network. In the beginning, all the sensor nodes receive the digital environmental reports generated by MCU by using the feature of multi-sensing utilities. As we have deployed the SDN controller, therefore the sensor nodes are self reprogrammable and can configure their preferences according to the application requirement. These deployed SDN controllers are programmed to check the characteristics of applications during the exchange of hello packets. In the meanwhile, the SDN controller sets a threshold for routing analytical operation based on nodes sensitivity. To this end, a random number is generated, which is then compared with the given threshold value chosen by the network administrator. All the sensor nodes produce the environmental reports according to this threshold value. As we have mentioned earlier that the proposed SADFIR supports the multi-application by self-reconfiguration; therefore, we deploy our network to sense three different parameters to get the environmental reports; temperature, pressure, and humidity. The following equations show the logical operations of application awareness based on the given threshold value:

(19) $$App-Thresh=\left\{\begin{array}{ccc} Temerture & If & Ran-Num<1stlevel\\ Pressure & If & 1stlevel<Ran-Num>2ndlevel\\ Humindity & If & Ran-Num>2ndlevel \end{array}\right.$$

**Algorithm 1:** Network settling phase of the proposed SADFIR routing protocol
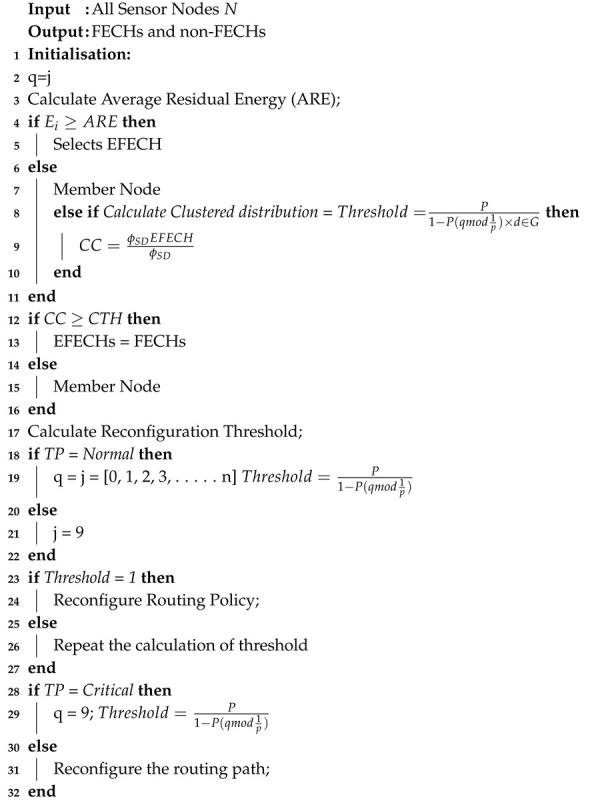



Similarly, the proposed SADFIR measures the critical data based on environmental attributes such as temperature and pressure and directly transmits this critical data immediately after sensing without any significant delay. It is attributed to the fact that the critical threshold value is set in the SDN controller, which is calculated by the following equation:

(20) $$App-Thresh=\left\{\begin{array}{ccc} Critical & If & Ran-Num<1stlevel\\ Temerture & If & 1stlevel<Ran-Num>2ndlevel\\ Pressure & If & Ran-Num>2ndlevel \end{array}\right.$$

**Figure 7:**
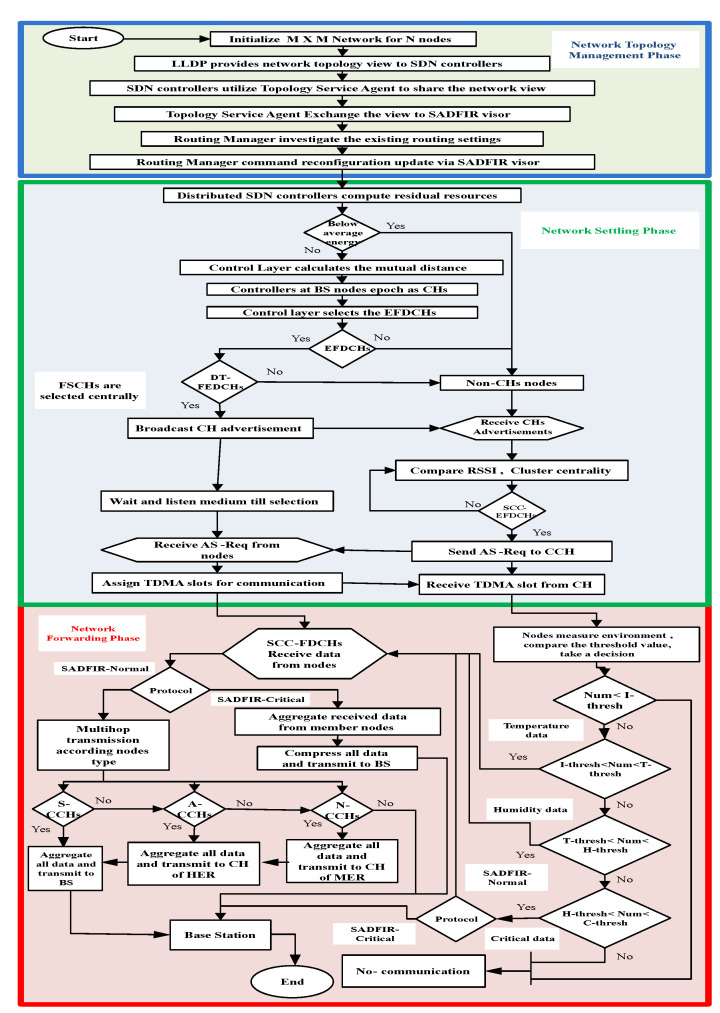
Flowchart of proposed model.

The proposed SADFIR is designed with a unique characteristic, where it utilizes the multi-path inter-cluster communication for all the existing types of environmental reports. Also, the proposed SADFIR is capable of self-trigger in case of critical data and enables transmission directly to avoid delay. These priorities of the threshold for application-specific reports are reconfigured through the SDN controller during the NSP. In contrast, NFP can also be called a transmission phase, which is mainly guided by the SDN controller at the control layer. The complete structure of the proposed model is summarized in the Flowchart, as shown in Figure 7.

## 6. Simulation Results and Discussion

In this section, we first briefly explain the simulation environment, and then we present the simulation results to show the achieved performance of the proposed model SADFIR. To this end, we consider network lifetime, network stability, and energy consumption ratio to measure the performance of the proposed models. To prove the significance of our outcomes, we conducted the same experiments for multiple times. We presented the average results in comparison with Advanced Centralized Energy Efficient Clustering (ACEEC) [32], Multi-Hop Centralized Energy Efficient Clustering (MCEEC) [33], Energy Efficient Scalable Routing Algorithm (EESRA) [34], and Energy-Balanced routing protocol (EBRP) [35] the state-of-the-art routing protocols. We conduct all of our experiments on the server with an Intel(R) Core(TM) CPU i5-8350U (1.7GHz) and 8GB of RAM. The SADFIR routing protocol is simulated in MATLAB environment.

In the simulation results, we analyze the performance on different scales of network parameters to identify the significance of the proposed model. In particular, we first consider various numbers of network areas, such as; 100 m × 100 m, 200 m × 200 m, 300 m × 300 m 600 m × 600 m, 800 m × 800 m, and 1000 m × 1000 m, in which we disperse 100 sensor nodes. In addition, to measure the performance based on residual energy of sensor nodes, we consider various numbers of initial energy levels, such as; 0.5,1.0,1.5,2.0,2.5, and 3.0 and disperse the 100 sensor nodes in the network areas described above, respectively. In the end, we also consider the impact of multiple numbers of sensor nodes dispersed in the 100m×100m network area and measure the number of packets received by the BS. In which, all the sensor nodes are connected with distributed multiple SDN controllers which reside at the control layer of BS. Meanwhile, BS is physically located at the top of the network. The rest of the simulation parameters are given in Table 2.

### 6.1. Network Lifetime and Network Stability

**Figure 8:**
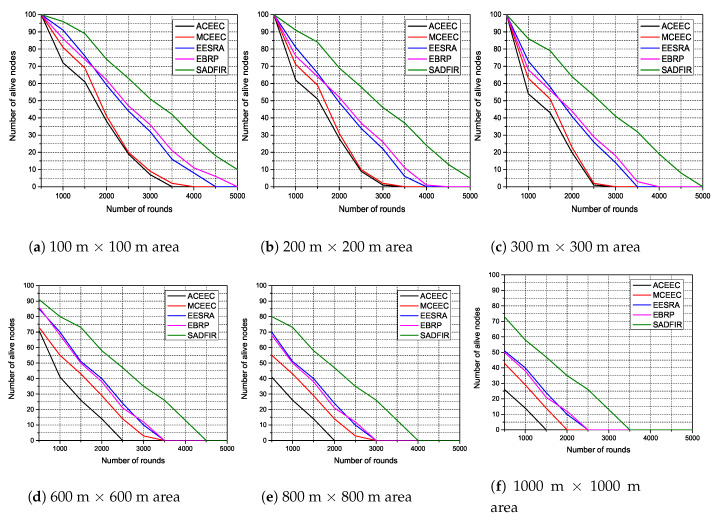
Energy Efficiency in terms of network lifetime in different scales of network area.

Network lifetime and network stability are the most important factors to measure the performance of any routing protocol in WSNs. Specifically, the network lifetime represents the total interval time from the start of the network until the death of all nodes. In contrast, network stability periods represent the total interval time from the start of the network until the death of the first node. In this subsection, we measure the network lifetime and network stability period and highlights the achieved performance through graphs. In Figure 8, we indicate the network lifetime and network stability period of the proposed model. In particular, we dispersed the 100 sensor nodes in the network areas of 100 m × 100 m, 200 m × 200 m, 300 m × 300 m 600 m × 600 m, 800 m × 800 m, and 1000 m × 1000 m and run the proposed model for 5000 communication rounds. The proposed model outperforms the state-of-the-art protocols and achieves the average of 43% and 13% better network lifetime and better network stability, respectively. The major reason behind this network efficiency is because the SDN controller develops the new routing scheme for each period and avoid the reconfigurations until the model produces the acceptable forwarding. Also, the forwarding phase of the proposed model controls the forwarding of identical information and wait until the new or critical information arrives.

### 6.2. Heterogeneity-Awareness and Network Scalability in Different Network Environment

**Figure 9:**
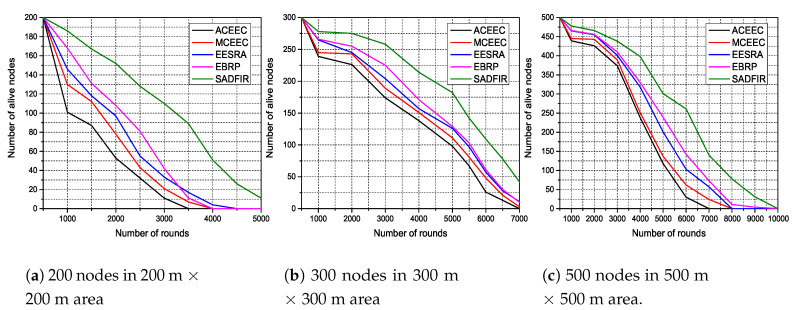
Energy Efficiency in terms of network lifetime with different numbers of sensor nodes in larger scales of network area.

In order to check the heterogeneity-awareness and scalability of the proposed SADFIR, we deploy higher numbers of sensor nodes in different scales of network areas. In particular, we deploy 200, 300 and 500 sensor nodes in the network area of 200 m × 200 m, 300 m × 300 m, 500 m × 500 m and runs the network for 5000, 7000 and 10,000 communication rounds respectively. In Figure 9, we present the heterogeneity-awareness of SADFIR in order to understand the network life-time and scalability of the network. In all the graphs of Figure 9, it is demonstrated that SADFIR outperforms the existing routing protocols and show better scalability for more large and dense IoT network scenarios. In particular, in Figure 9a, the sensor nodes in SADFIR are still alive until the end of network time, while the sensor nodes in other routing protocols are dead far before SADFIR. Similarly, in Figure 9b, EESRA and EBRP perform significantly, but still, SADFIR outperforms all of them and remain active until the end of network time. In Figure 9c, SADFIR outperform the EBRP by 810 communication rounds, whereas all the other routing protocols exhaust their total energy before EBRP routing protocol. The reason behind this efficient performance of SADFIR is because the multiple distributed SDN controllers avoid the reconfiguration time and forward the packets with a minimum rate of communication energy. In addition, the SDN controller sends only critical data, therefore higher number of sensor nodes favour the SADFIR and subsequently enhance the communication efficiency. These results indicate the better heterogeneity-awareness and scalability once the density and network area is increased.

### 6.3. Residual Energy of Sensor Nodes

**Figure 10:**
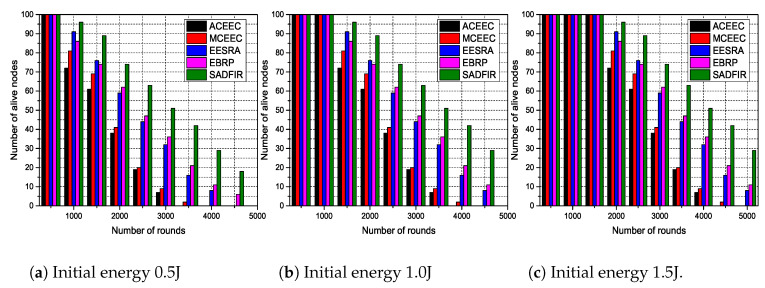

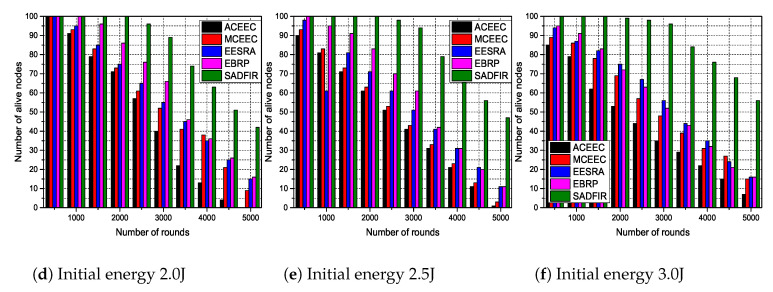
Number of alive nodes on different values of energy levels on different scales of network area.

The dispersed sensor nodes are supposed to transmit the critical data continuously to the BS, therefore higher levels of the initial energy of sensor nodes can prolong the network lifetime. This initial energy initializes the network and helps in selecting the CHs in the first epoch. These CHs consume the energy to communicate with the BS. Hence, the residual energy of sensor nodes is to be considered to select the CHs in the next epoch. Whereas, the higher energy require the bigger size of the battery, and due to the matter of the fact that the sensor nodes are supposed to be deployed in the critical network, therefore we cannot set the initial energy to the higher numbers. To this end, in this paper, we consider the real-time applications of WSNs and set considerable amounts of initial energy to the sensor nodes. In Figure 10, we test the network lifetime of the proposed model in different scales, where we set the initial energy to 0.5,1.0,1.5,2.0,2.5 and 3.0 and disperse the 100 sensor nodes in the 100 m × 100 m, 200 m × 200 m, 300 m × 300 m, 600 m × 600 m, 800 m × 800 m, and 1000 m × 1000 m network areas, respectively and run the model for 5000 communication rounds. In this Figure 10, ACEEC performs worst because of the single-hop communication and central control of route selection whereas, EBRP tried to compete the proposed SADFIR in the first two graphs but failed to prolong on the bigger scale of networks. Figure 10 proves that the proposed model SADFIR produces the optimal results not only in conventional settings of WSNs but also in the bigger scale of networks. This is due to the matter of fact that in the proposed SADFIR, the multiple distributed SDN controllers help the sensor nodes to adopt reprogrammable configurations with an understanding of residual energy levels.

### 6.4. Energy Consumption Ratio

**Figure 11:**
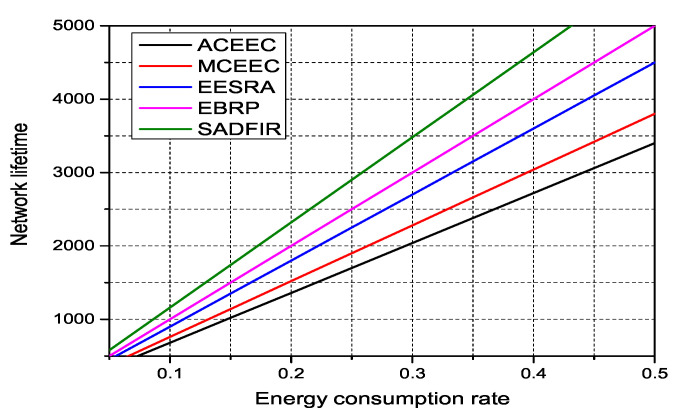
Energy consumption rate as network operation progress in network of 100m×100m with 100 nodes.

The energy consumption ratio is considered to measure the overall network performance of any routing protocol. As the network operation tends to progress, the sensor nodes keep consuming the energy to perform the assigned tasks. In the traditional WSNs routing protocols, the initial energy of sensor nodes is set to 0.5 Joule. Therefore, we consider testing our proposed model SADFIR for energy consumption ratio. To this end, in Figure 11, we set the initial energy of all the 100 sensor nodes to 0.5 J in all the routing protocols and run the protocols for 5000 communication rounds. In Figure 11, it is demonstrated that all the models continuously consumes the energy with an increasing number of communication rounds, where the network of ACEEC, MCEEC, and EESRA dies after the 3346, 3804 and 4509 communication rounds. Whereas, the EBRP and SADFIR continuously running till the end of the network settings, where we can see that the proposed SADFIR has a minimum energy consumption graph, which results in better performance than EBRP routing protocol.

### 6.5. Packets Delivery to the BS

**Figure 12:**
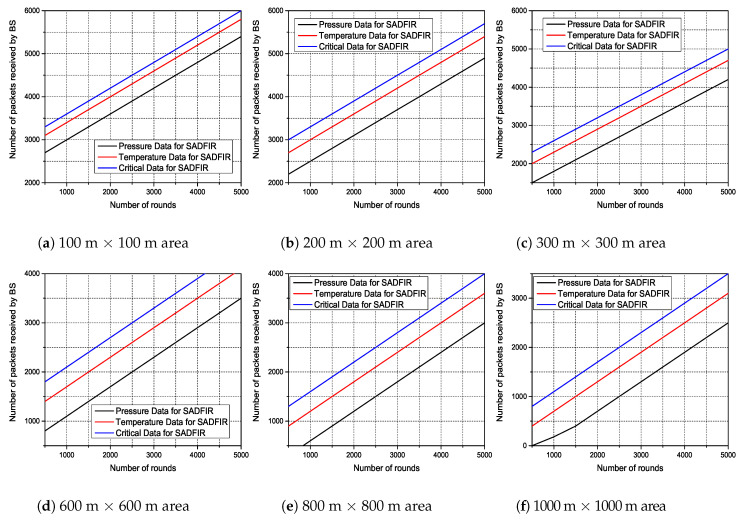
Multi-type data received at BS over network operations in different numbers of network scales.

As we have mentioned in the earlier sections, the routing pattern of the proposed model SADFIR is based on the distributed environment and the SDN controller driven. To this end, we consider multiple applications of environmental parameters that are supposed to be sensed and transmitted by the sensor nodes. Therefore, the final transmitted packets by the sensor nodes contain the sensed data of multiple environments as shown in Figure 12. To check the efficiency of the proposed SADFIR in multiple environments, we disperse the 100 sensor nodes in the 100 m × 100 m, 200 m × 200 m, 300 m × 300 m, 600 m × 600 m, 800 m × 800 m, and 1000 m × 1000 m network areas, respectively and run the model for 5000 communication rounds as considered in the previous results. In Figure 12, the proposed SADFIR achieves significant performance according to the given threshold from the central SDN controller. In all the graphs of Figure 12, it is demonstrated that in every scenario of the network size, the amounts of different type of information are different and it is achieved through the application preference which keeps resetting by the SDN controller in each graph. Therefore, the reconfigurations in the proposed SADFIR helps to bring flexibility to receive the required application data for the particular interval of time from a single sensor node.

## 7. Conclusions

In this paper, we propose the SDN-enabled distributed SADFIR clustering routing protocol for IoT network devices with the distinct features of heterogeneity-awareness and application-awareness. The programable OpenFlow equipped IoT devices are regionally distributed at the infrastructure layer and continuously coordinate with the above control and application layer to carry out periodic reconfiguration during the network operations. The distributed multiple SDN controllers at the control layer perform mutual coordinations to share and optimize the computational performance of SADFIR settling phase and enforce the routing paths at the infrastructure layer to IoT devices. The proposed SADFIR routing protocol is executed in our designed Distributed SDN-enabled Iteratively Reconfigurable Internet of Things (DSDN-IRIoT), which follows the layer-wise approach of the network operation. Furthermore, the dynamic reconfiguration capabilities of the proposed SADFIR routing protocol are the source of additional performance optimization. In order to prove the performance of the proposed SADFIR, we provide extensive simulation level experiments in terms of network lifetime, network stability, energy consumption ratio, application-aware and heterogeneity-aware. In all the experiments, the proposed SADFIR outperforms the state-of-the-art routing protocols with significant margins.

## Figures and Tables

**Table 1:** Summarized review of SDN-based sensor networks.

Management Architecture	Network Scalability	Application Awareness	Heterogeneity Awareness	Topology Discovery	Computational Cost	Periodic Reconfiguration
SDN-WISE [1]	✓	✓	✓	✓		✓
SDCSN [7]	✓		✓		✓	
Sensor OpenFlow [13]	✓		✓		✓	
Soft-WSN [21]	✓				✓	
TinySDN [22]		✓	✓	✓		✓
TinySDM [30]			✓		✓	✓
SDWN [31]	✓	✓				✓
ACEEC [32]	✓				✓	
MCEEC [33]		✓			✓	
EESRA [34]	✓		✓	✓		
EBRP [35]			✓		✓	
SADFIR	✓	✓	✓	✓	✓	✓

**Table 2:** General simulation parameters.

Parameter	Value
Network size	various (described result-wise)
Initial Energy	various (described result-wise)
Network nodes	various (described result-wise)
Percentage of CHs	10%
Data Packet size	4000 bit
Transmit amplifier (Eamp)	100 pj/bit/m2
Data Aggregation Energy cost	50pj/bit J
Transmitter Electronics (EelectTx)	50 nJ/bit
Receiver Electronics (EelecRx)	50 nJ/bit

## References

[1] Galluccio L., Milardo S., Morabito G., Palazzo S. SDN-WISE: Design, prototyping and experimentation of a stateful SDN solution for WIreless SEnsor networks. Proceedings of the 2015 IEEE Conference on Computer Communications (INFOCOM).

[2] Heinzelman W., Chandrakasan A., Balakrishnan H. Energy-efficient communication protocol for wireless microsensor networks. Proceedings of the 33rd Annual Hawaii International Conference on System Sciences.

[3] Asad M., Aslam M. Heuristic Path-Reconfiguration Algorithm Using Multi-hop Opportunistic Routing in WSNs. Proceedings of the 2018 International Conference on Frontiers of Information Technology (FIT).

[4] Heinzelman W., Chandrakasan A., Balakrishnan H. (2002). An application-specific protocol architecture for wireless microsensor networks. IEEE Trans. Wirel. Commun..

[5] Garcia F.P., de Souza J.N., Andrade R.M. An energy-efficient passive monitoring system for wireless sensor networks. Proceedings of the Sustainable Internet and ICT for Sustainability (SustainIT).

[6] Keller L., Atsan E., Argyraki K., Fragouli C. (2013). SenseCode: Network coding for reliable sensor networks. ACM Trans. Sens. Networks (TOSN).

[7] Olivier F., Carlos G., Florent N. Sdn based architecture for clustered wsn. Proceedings of the 2015 9th International Conference on Innovative Mobile and Internet Services in Ubiquitous Computing.

[8] Ndiaye M., Hancke G.P., Abu-Mahfouz A.M. (2017). Software Defined Networking for Improved Wireless Sensor Network Management: A Survey. Sensors.

[9] Aslam M., Hu X., Wang F. (2017). Sacfir: Sdn-based application-aware centralized adaptive flow iterative reconfiguring routing protocol for wsns. Sensors.

[10] Gupta A., Vanbever L., Shahbaz M., Donovan S.P., Schlinker B., Feamster N., Rexford J., Shenker S., Clark R., Katz-Bassett E. (2015). Sdx: A software defined internet exchange. ACM SIGCOMM Comput. Commun. Rev..

[11] Agarwal S., Kodialam M., Lakshman T. Traffic engineering in software defined networks. Proceedings of the 2013 Proceedings IEEE INFOCOM.

[12] Costanzo S., Galluccio L., Morabito G., Palazzo S. Software Defined Wireless Networks: Unbridling SDNs. Proceedings of the European Workshop on Software Defined Networking.

[13] Luo T., Tan H.P., Quek T.Q. (2012). Sensor OpenFlow: Enabling software-defined wireless sensor networks. IEEE Commun. Lett..

[14] Fontes R.R., Afzal S., Brito S.H., Santos M.A., Rothenberg C.E. Mininet-WiFi: Emulating software-defined wireless networks. Proceedings of the 2015 11th International Conference on Network and Service Management (CNSM).

[15] De Gante A., Aslan M., Matrawy A. Smart wireless sensor network management based on software-defined networking. Proceedings of the 2014 27th Biennial Symposium on Communications (QBSC).

[16] Yu H., Jia Z., Ju L., Liu C., Ding X. Energy Efficient Routing Algorithm Using Software Defining Network for WSNs via Unequal Clustering. Proceedings of the International Conference on Geo-Informatics in Resource Management and Sustainable Ecosystems.

[17] Kahjogh B.O., Bernstein G. Energy and latency optimization in software defined wireless networks. Proceedings of the 2017 Ninth International Conference on Ubiquitous and Future Networks.

[18] Li M., Zhao L., Liang H. (2017). An SMDP-based Prioritized Channel Allocation Scheme in Cognitive Enabled Vehicular Ad Hoc Networks. IEEE Trans. Veh. Technol..

[19] Kocakulak M., Butun I. An overview of Wireless Sensor Networks towards internet of things. Proceedings of the 2017 IEEE 7th Annual Computing and Communication Workshop and Conference (CCWC).

[20] Hu F., Hao Q., Bao K. (2014). A survey on software-defined network and openflow: From concept to implementation. IEEE Commun. Surv. Tutors.

[21] Bera S., Misra S., Roy S.K., Obaidat M.S. (2018). Soft-WSN: Software-Defined WSN Management System for IoT Applications. IEEE Syst. J..

[22] De Oliveira B.T., Gabriel L.B., Margi C.B. (2015). TinySDN: Enabling multiple controllers for software-defined wireless sensor networks. IEEE Lat. Am. Trans..

[23] Zhou J., Jiang H., Wu J., Wu L., Zhu C., Li W. (2016). SDN-based application framework for wireless sensor and actor networks. IEEE Access.

[24] Xiong X., Hou L., Zheng K., Xiang W., Hossain M.S., Rahman S.M.M. (2016). Smdp-based radio resource allocation scheme in software-defined internet of things networks. IEEE Sens. J..

[25] Ejaz W., Naeem M., Basharat M., Anpalagan A., Kandeepan S. (2016). Efficient wireless power transfer in software-defined wireless sensor networks. IEEE Sens. J..

[26] Misra S., Bera S., Achuthananda M., Pal S.K., Obaidat M.S. (2017). Situation-aware protocol switching in software-defined wireless sensor network systems. IEEE Syst. J..

[27] Al-Janabi T.A., Al-Raweshidy H.S. Efficient whale optimisation algorithm-based SDN clustering for IoT focused on node density. Proceedings of the 2017 16th Annual Mediterranean Ad Hoc Networking Workshop (Med-Hoc-Net).

[28] Han L., Sun S., Joo B., Jin X., Han S. (2016). QoS-aware routing mechanism in OpenFlow-enabled wireless multimedia sensor networks. Int. J. Distrib. Sens. Netw..

[29] Pradeepa R., Pushpalatha M. SDN enabled SPIN routing protocol for wireless sensor networks. Proceedings of the 2016 International Conference on Wireless Communications, Signal Processing and Networking (WiSPNET).

[30] Cao C., Luo L., Gao Y., Dong W., Chen C. TinySDM: Software defined measurement in wireless sensor networks. Proceedings of the 15th International Conference on Information Processing in Sensor Networks.

[31] Hu H., Chen H.H., Mueller P., Hu R.Q., Rui Y. (2015). Software defined wireless networks (SDWN): Part 1 [guest editorial]. IEEE Commun. Mag..

[32] Aslam M., Munir E.U., Rafique M.M., Hu X. (2016). Adaptive energy-efficient clustering path planning routing protocols for heterogeneous wireless sensor networks. Sustain. Comput. Inform. Syst..

[33] Javaid N., Aslam M., Ahmad A., Khan Z.A., Alghamdi T.A. MCEEC: Multi-Hop Centralized Energy Efficient Clustering Routing Protocol for WSNs. Proceedings of the 2014 IEEE International Conference on Communications (ICC).

[34] Elsmany E.F.A., Omar M.A., Wan T.C., Altahir A.A. (2019). EESRA: Energy Efficient Scalable Routing Algorithm for Wireless Sensor Networks. IEEE Access.

[35] Ren F., Zhang J., He T., Lin C., Ren S.K.D. (2011). EBRP: Energy-balanced routing protocol for data gathering in wireless sensor networks. IEEE Trans. Parallel Distrib. Syst..

[36] Gude N., Koponen T., Pettit J., Pfaff B., Casado M., McKeown N., Shenker S. (2008). NOX: Towards an operating system for networks. ACM Sigcomm Comput. Commun. Rev..

[37] Pakzad F., Portmann M., Tan W.L., Indulska J. Efficient topology discovery in software defined networks. Proceedings of the 2014 8th International Conference on Signal Processing and Communication Systems (ICSPCS).

[38] Krishnan S., Yegin A., Montavont N., Njedjou E., Veerepalli S. (2007). Link-Layer Event Notifications for Detecting Network Attachments. https://tools.ietf.org/html/rfc4957.

[39] O’Shea D., Cionca V., Pesch D. The Presidium of Wireless Sensor Networks-A Software Defined Wireless Sensor Network Architecture. Proceedings of the International Conference on Mobile Networks and Management.

